# Health-Related Social and Ethical Considerations towards the Utilization of Dental Medical Services by Seniors: Influencing and Protective Factors, Vulnerability, Resilience and Sense of Coherence

**DOI:** 10.3390/ijerph18042048

**Published:** 2021-02-19

**Authors:** Ina Nitschke, Sebastian Hahnel, Julia Jockusch

**Affiliations:** 1 Clinic for Prosthetic Dentistry and Dental Materials Science, Leipzig University Medical Center, 04103 Leipzig, Germany; ina.nitschke@medizin.uni-leipzig.de; 2 Clinic of General, Special Care and Geriatric Dentistry, Center of Dental Medicine, University of Zurich, 8032 Zurich, Switzerland; julia.jockusch@zzm.uzh.ch

**Keywords:** uptake of dental services, resilience, coherence, salutogenesis, oral healthcare research, ageism

## Abstract

The aim is to analyze protective and modifying factors (e.g., vulnerability, resilience, sense of coherence) in relation to the utilization of dental services by seniors at different levels of the healthcare system. Terminological imprecision in the use and transfer of existing terms (sense of coherence, resilience, salutogenesis) to gerodontology is clarified. Factors influencing a reduced utilization (static/dynamic factors) can occur isolated or in combination and, thus, model the risk of a reduced utilization of dental services (influencing-factor mechanism). Protective factors of utilization include patient-specific factors for self-motivation and factors that promote oral-health-related resilience. Resistance forces that counteract can be identified as oral-health-related resilience factors. Achieving social and individual appreciation and establishing a prevention-oriented approach to utilization will be increasingly challenging, since the population is becoming older and access is not equal in terms of opportunity. Resistance forces need to be strengthened in an ethical context. Studies should increasingly present resilience processes, determinants and modes of action at the various interfaces in the healthcare system, which can ensure sustainable medical care in old age. The concepts conveyed here are generally valid and able to point out inequalities and ageism in access to dental services.

## 1. Introduction

The utilization behavior of medical services cannot be explained by a single factor. A behavioral model for the use of health services was published by Andersen in the late 1960s and has been continuously developed since then [1,2,3]. This model can be used to describe a broad spectrum of possible influencing factors on outpatient utilization. The influencing factors on the utilization of medical services are called “predisposing characteristics“, which are characteristics of a person that indirectly affect utilization (e.g., demo-graphic characteristics, social structure, health beliefs), the “enabling resources” (prerequisites for utilization, distinction between personal, family, community resources) and “need” (distinction between needs that are subjective, perceived by the patient and objective, evaluated needs by the physician) [2].

In regard to dentistry, it is known that the utilization of dental services decreases with increasing age. The utilization of medical services increases meanwhile [4]. The reasons for a reduced utilization are as diverse as the heterogeneous group of older people themselves. In addition to objectively assessable influencing factors (e.g., socio-demographic [5,6,7], socio-economic [6,7] or health factors [5,6]), subjective reasons of older people are also decisive for their utilization of dental services [5]. From the experience of gerostomatologists, it is possible to identify patterns and cause–effect relationships of changeable and unchangeable (influencing) factors that can reduce or even prevent the utilization. A variety of events (e.g., hospital admissions, loss of close relatives, transition to a care situation) and life circumstances (e.g., a decrease in personal self-responsibility, reduced mobility [5] and increasing morbidity [5], poverty) can influence the type and frequency of dental healthcare at the patient level [8]. Ensuring equal opportunities and equity for older people in access to dental healthcare services poses challenges for dentists and their teams. If ethical principles such as justice, the principle of non-maleficiency and beneficence and respect for the patient’s autonomy are to be applied [9], dentists working in the field of gerodontology are often faced with ethical dilemmas [10].

The present work examines the individual and structural factors of the utilization behavior of older people with the example of gerodontology. Both the field of dentistry and the chosen patient group (old and very old people, 65 years of age and older) are well defined, and it is assumed that almost every reader, in contrast to other medical specialties, has an own experience with dental healthcare and can, therefore, understand the thoughts of the expert panel in a sustainable way. The explanations are mainly based on the core concept of the model described by Andersen [3]. Furthermore, the aim is to analyze protective factors as well as modifying factors (vulnerability, resilience, sense of coherence) in relation to the utilization of dental services by older people. The overall objective of this work is to propose a new wording of the existing terms for the general usage in gerodontology and gerodontological research. Additionally, proposals for a strategy for the implementation of a supportive resilience structure are made.

## 2. Materials and Methods

A selective literature search based on the identification of factors influencing the uptake of dental services by seniors in databases (e.g., PubMed, ScienceDirect), supplemented by a free internet search as well as a search in grey literature is the basis for the present work. Due to the lack of literature on the topic in the field of gerodontology, specific inclusion and exclusion criteria for the analyzed literature have not been established. The selective literature review, however, was conducted with respect to the patient population resulting from the gerodontology field of work. The results of the work should be applicable to the selected patient group, which includes old or very old people (65 years and older). Since cultural as well as socio-political aspects can also play a role in this analysis, the expert panel attempted to highlight all aspects objectively in order to enable an international interpretation of the results.

In a first step, a structured literature search based on the PICO system [11] to identify factors was conducted. Literature in English and German published between 1965 and 2020 was screened. The search terms included “uptake of dental services OR utilization of dental services OR usage of dental services AND elderly OR old* OR aged” with a combination of one of the terms of interest, i.e., “resilience, sense of coherence, salutogenesis, and vulnerability”.

In a second step, the expert panel then utilized this literature overview as a working definition of utilization of dental services influencing factors and ethical aspects to deepen or extend the obtained results by thesis of the expert panel (including opinions, experiences and ethical analysis). This expert panel consisted of the authors with long-term, university-based clinical and scientific experience in the field of gerodontology. Two of the three experts experience two very differently structured healthcare systems regarding dental care. All members of the expert panel are certified specialists of the German Society (Deutsche Gesellschaft für Alterszahnmedizin) for the field of gerodontology. In a third step, based on the selective literature search in combination with the expert panel, influencing factors and protective factors of utilization of dental services by older people are assigned to the levels of the healthcare system (macro, mesio and micro level). Based on the developed influencing factors, the expert panel intended to create an influencing-factor mechanism of the utilization of healthcare services. In addition, in a fourth step, a terminological analysis was done with regard to the utilization of the terms sense of coherence, resilience and salutogenesis in the field of gerodontology. The bidirectional transfer of terms from different scientific fields can lead to terminological inaccuracies in the application. An attempt has been made to adapt the terms correctly for the gerodontological field.

## 3. Results

### 3.1. Results of the Selective Literature Search and Expert Panel

#### 3.1.1. Influencing Factors for Non-Utilization of Dental Services by Older People

A number of influencing factors for the utilization or non-utilization of dental healthcare services have been identified in the literature—mostly at the micro level (individual patient–physician level) [5,6,7,8,12,13,14,15,16,17,18,19,20,21,22,23,24,25] (Table 1, part “Micro Level”).

Next to well-evaluated factors such as general and oral health status [5,6,7,8,12,13,14,15,16,17,18,19,20,21,22,23,24,25,26,27,28,29,30,31,32,33,34], health determinants [5,6,7,8,12,13,14,15,16,17,18,19,20,21,22,23,24,25,26,27,28,29,30,31,32,33,34] and socio-demographic/-economic factors [5,6,7,8,12,13,14,15,16,17,18,19,20,21,22,23,24,25,26,27,28,29,30,31,32,33,34], evidence for subjective, rational reasons and factors (micro level, patient side) are only insufficiently described in the literature.

An important factor is the presence of fears (e.g., dental phobia, injection phobias) [16,17]. A refusal due to fear and misunderstanding can also be the result of depression, delirium or any other disorder that affects the patient’s ability to decide to seek dental and medical help.

The individual right to neglect [21] and the refusal to adapt to social standards by a patient, or the denial of school dentistry or a loss of confidence in this [22] should be mentioned here.

Furthermore, it is possible that patients do not undergo treatment due to financial restrictions [17,19] but feel ashamed to admit this publicly in front of the dentist or the dental team.

The patient’s trust in the physician as a service provider was also strained when, for example, a law to combat corruption in the health sector was passed in Germany in 2016. Criminal offences such as “bribery in the healthcare sector” (§ 299a and b StGB) were introduced, which can also lead to irritation and then non-use by patients [24].

To what extent self-perception of the need for treatment plays a role in the utilization process is unclear. However, it was shown that older patients subjectively assess their need for treatment less than this is objectively evaluated by the dentist [12,17,18].

Another possible subjective reason for not using dental services could be a subjective opinion that the cost/risk for preventive efforts is too high. Reasons for this might be the following aspects. First of all, in most countries, there are costs for dental treatment as opposed to medical services, which are provided by health insurances. So, the individual question arises, how much the health of the tooth is valued for each individual. Second, the question arises if the (own) costs for prevention justify the prevention of possible oral diseases, or can costs really be avoided?

At the micro level of healthcare providers and their teams, it has to be mentioned that negative social experiences in dealing with older people last longer than positive experiences [35]. Baltes and Silverberg (1994) describe inappropriate and dysfunctional support with reluctantly provided social support as a risk factor resulting in the medical team avoiding this situation [25]. Factors in the patient–dentist relationship or within the infrastructure or the dental team and their skills are assumable but have not been described very often until now (Table 1, part “Micro Level”).

#### 3.1.2. Vulnerability and Resilience in Older People

The terms vulnerability and resilience are used in different contexts in quite different ways. In medicine, when assessing the vulnerability of older people, in addition to their medical needs, psychological and sociological aspects are particularly relevant. Here, the term is used to describe a group of people. Vulnerability also refers to the individual predisposition to reduced resistance to stress, which favors the occurrence of a disorder or disease when the individual is exposed to certain stimuli or circumstances.

Schröder-Butterfill and Marianti (2006) describe a concrete theoretical framework for vulnerability in old age, whereby this framework is composed of the risks of a person being exposed to a certain threat, that the threat will become real and that this person will not be able to defend oneself independently against the threat [36].

In geriatrics, the term frailty is often used instead of vulnerability. In the geriatric context, frailty means “the vulnerability of (very) old people to endogenous and, above all, exogenous stressors that are able to destabilize a finely balanced–but fragile–system” [37]. A vulnerable patient group is, therefore, a group that is especially in need of protective interventions due to its specific (medical, cognitive or social) characteristics.

Resilience (the capacity to resist and adapt; originally: psychological resistance; Latin: resilire: to bounce back, bounce off) describes the capacity of a person to resist a threat (e.g., crises, failures, burdens, loneliness due to loss of partner or illness) [38], the ability “to stabilize the self-concept even when facing massive burdens” [39] and also to have a regenerative effect on the effects of the threats [38] as well as to learn from these experiences and to deal with them well (further development of the self-concept; coping). Resilience allows a person to maintain or restore one’s personal stability (structure, functionality, health, etc.) even under adverse events or circumstances. The basis for this is the possibility of being able to use personal as well as socially provided resources. Resilience means that after a reduction in performance, the initial level can be achieved again. Old and very old people are exposed to structural risk factors (dynamic and static influencing factors, e.g., financial situation, transport situation, offer of mobile outreach services, etc.; see also Table 1), which may reduce the utilization of dental healthcare services, as they cannot change or overcome these factors on their own due to their vulnerability/frailty.

#### 3.1.3. Salutogenesis and the Sense of Coherence—A Different Approach

The importance of salutogenesis increases with decreasing utilization of dental healthcare services with increasing age [4,40]. Salutogenesis [41] is the process of the “development of health”. Since it is a process that is based on the human being as a bio-psycho-social unit, the term “convalescence of the oral cavity” is more appropriate than the term oral health. In contrast to the identification of risk factors that promote disease, the concept of salutogenesis is focused on the question of circumstances for health development [42,43]. Depending on the individual risk profile, psycho-social salutogenic factors can be considered to increase recovery. These include so-called personal determinants (cf. mesio level; Table 2) such as the concept of resilience and sense of coherence. Furthermore, contextual determinants can be identified. These relate primarily to concepts of social integration and social support (cf. Table 3). At the mesio level, the responsibility for the provision of welfare by both the government and health insurers should also be mentioned here. However, factors such as income available to the household, social status and religious affiliation are also defined as factors with an influence on salutogenesis [44].

The concept of sense of coherence, on the other hand, is based on the “way of looking at the world”, i.e., the personal attitude towards the world [41]. It describes the possibility of understanding events and the ability to put the meaning of events into the context of one’s own life [41]. The sense of coherence, thus, enables people to activate and use resources that increase their resistance to stressors (or specific pathogeneses) [43].

Eriksson and Mittelmark (2017) describe the sense of coherence as “a mixture of optimism combined with a sense of control” [45]. According to them, the sense of coherence consists of three components. First, comprehensibility, which allows internal and external stimuli/stressors to be rational understandable. Secondly, manageability, which explains the feeling that one is able to deal with the stimuli because of resources (e.g., social service, care staff, family or friends) that are at disposal. This component has to do with the ability to cope and the willingness to solve problems. Thirdly, meaningfulness is a motivational aspect, which might become important when facing challenges or problems [45].

Antonovsky concluded that individuals who have sufficient generalized resistance resources form a strong sense of coherence [41]. A person “equipped” in this way will find it easier to seek or train new resources. They will become more and more resistant to stressors and, thus, maintain and promote their health [42].

Reversely, this also means that this resistance force, when it decreases, limits the ability to protect oneself from disease using one’s own resources. Life experiences, which older people usually have at their disposal in sufficient quantities, are characterized by a balanced relationship between over- and underchallenging, and they convey the feeling that one can contribute something to the outcome. With increasing frailty, there will no longer be a balanced relationship; the individual faces everyday things, which he or she has experienced and lived well up to now, more and more as a daily excessive demand and must accept that his or her resistance decreases. Here, others should provide resources or support for the frail.

#### 3.1.4. Life Events in Old Age as a Risk Factor

Sustainable events in old age (e.g., the loss of a partner or other social contacts, changes in general well-being due to external circumstances, giving up the home and moving into a long-term care facility, etc.) may have additional negative effects (e.g., through changes in cognition, promotion of depression, etc.) on the regular age-physiological changes in the human organism. These physical changes triggered by events in turn can lead to a reduced availability of protective resources. The human being is able to react to these situations—in different individual ways (sense of coherence, resilience). To support this optimization process, appropriate techniques and interventions from the field of geronto-psychology should be used [46].

The manageability aspect of Antonovsky’s salutogenesis concept does not have to be a resource under the control of the increasing frailty, but a legitimate other—i.e., spouse, children, grandchildren, relatives or even a dentist—can provide this resource. This “delegating responsibility” to legitimate others distinguishes Antonovsky’s approach, for example, from the “locus of control” often used in medical psychology [42] and should be consciously perceived by the representatives of the other levels (cf. Table 1).

### 3.2. Analysis of the Literature Search Conducted by the Expert Panel

#### 3.2.1. Factors Influencing the Use of Dental Services by Older People at the Various Levels of the Healthcare System and Their Influence Factor Mechanism

The factors influencing the utilization of dental care by old people identified in the literature (cf. Section 3.1.1, Table 1) are mostly factors on the micro level (individual patient–physician level). Patient- or dentist-independent factors at the level of the healthcare system at the macro level (social-political level) and mesio level (social level), other influencing factors at the patient level (subjective, rational reasons and objective influencing factors) as well as other factors at the level of the healthcare providers and their teams (micro level) are lacking. The expert panel has tried to assign the factors mentioned in the literature and factors identified by the expert panel to the various levels of the healthcare system (macro, mesio, micro level). The influencing factors, which become risk factors for a reduced utilization of dental healthcare services due to occurring changes, have to be classified into static not individually influenceable and dynamic, easily changeable factors. These occur either isolated or in combination, whereby they reinforce or benefit each other (Table 1).

**Figure 1:**
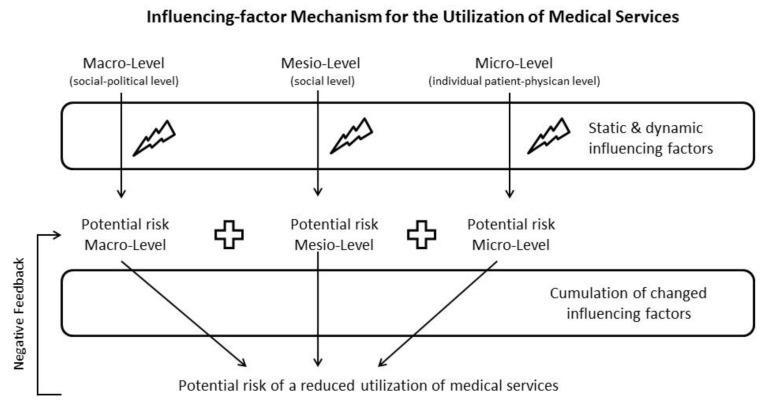
Influencing-factor mechanism of the utilization of healthcare services.

A single factor per se does not necessarily lead to a reduced utilization. There is, however, an influencing-factor mechanism that indicates a process, which may result in a reduction in utilization (Figure 1). The influencing-factor mechanism is applicable to all healthcare services, including dentistry.

#### 3.2.2. Protective Factors of the Utilization of Dental Healthcare Services

In contrast to the influencing factors, which may also become risk factors for the non-utilization of dental healthcare services, the expert panel differentiated protective factors that favor the resumption or continuation of the utilization of dental healthcare services. The protective factors are assigned to three protective levels. These include:

- Individual level;
- Social level;
- Social-political level.

These levels include patient-specific factors for self-motivation (Table 2) and factors that promote and strengthen oral-health-related resilience (Table 3), which may contribute to the improvement of a control-oriented utilization of dental care.

### 3.3. Development of a New Terminology with Regard to Gerodontology

Terminological analysis was done with regard to the utilization of the terms sense of coherence, resilience and salutogenesis in the field of gerodontology. Furthermore, based on the results of the literature review, the expert panel attempted to develop a new terminology of the abovementioned terms in relation to gerodontology, which, incorporating a medical–ethical point of view, aims at their use in practical every day and research issues in the field of gerodontology.

The general resilience of a person includes utilization factors for typical challenges faced by an older person (e.g., how to ensure that the patient continues to have access to the dental office even if mobility is reduced or the prejudice “at my age it is no longer worth visiting the dentist” is dispelled?). Special resilience factors refer to risks that are present in certain phases of life and in the simultaneous presence of certain non-normative risk factors (e.g., poor oral health with reduced awareness of the treatment need and an overall pessimistic perception of one’s life phase).

In the context of the utilization of dental healthcare services, all adjustments/factors or developments, both of the older person and their close environment and the external system, are summarized under the term resilience (i.e., accumulation of risk-minimizing resources) in consideration of the risk of a reduced utilization of dental healthcare services influencing the salutogenesis of the (older) person. Achieving the complete, smooth and interlocking functioning of all resilience factors is very difficult, since it is a dynamic process. Furthermore, the factors can act indirectly via a protective buffer function or by directly promoting a certain development. On the other hand, their absence may also result in a development risk. Individual factors have the potential to add up and mutually reinforce each other. With regard to the context, a distinction between general and differential protective factors is required. The former is situation-independent.

Furthermore, the expert panel suggests that all resistance forces that counteract a reduced utilization of dental healthcare services and that should be strengthened in an ethical context may also be called oral-health-related resilience factors. These aim at maintaining the best possible quality of life based on sustainable convalescence of the oral cavity and, consequently, sustainable oral health. Oral-health-related resilience factors should be promoted by ensuring lifelong access to dental care throughout the different life situations in the different phases of life until death. From the dentist’s point of view, a resilient older person with regard to oral health is the patient who maintains the utilization of dental services despite health-related, structural, social and financial changes in living conditions (i.e., despite the existing risks of a reduced utilization).

In old age, a distinction between different resiliencies associated with utilization can be made. These include (a) resiliency, which comes into effect after a discontinuation of control-oriented dental care, (b) resiliency in connection with an acute process (emergency-oriented use, e.g., after abscess, trauma) and (c) resiliency, which is defined by a negative profit–loss balance due to impairing events such as loss of health and social structures (prevention-oriented use, e.g., check-ups with no curative therapeutic purpose in cases of advanced dementia in order to avoid or detect oral-health-related problems).

## 4. Discussion

### 4.1. Discussion of Findings

#### Influencing and Protective Factors

Influencing and protective factors for utilization were developed. Not all findings are evidence-based, since clinical studies on the topic, especially on subjective, rational factors of patients, have been insufficiently or not at all investigated.

Regarding the influencing factors for non-utilization of dental services, most of the reported factors rarely consider the dentists’ and their dental teams’ level of influence nor the patients’ subjective point of view but primarily focus objectively on the older person and their specific influencing factors. Therefore, they reveal a rather paternalistic view on the utilization of dental services. The patients’ subjective, rational reasons and factors for a non-utilization of dental services also need to be considered to provide a detailed description of all influencing factors.

Risk factors of a reduced utilization with influence on the development of a disease allow only a pathogenetic approach [43]. It is shown that a deterioration of oral health can be expected due to reduced utilization of dental healthcare services. The results of the Fifth German Oral Health Study (Fünfte Deutsche Mundgesundheitsstudie (DMS V)) proved this for dentistry dependent of the health awareness (control-oriented vs. complaint-oriented utilization of the dentist) [47]. In the case of a control-oriented approach, seniors (65–74 years of age and older, 75–100 years of age) showed a lower DMF/T (decay, missing, filled teeth) value and fewer extracted teeth than patients who only visited the dentist for complaints [47,48]. It was also shown that people in need of care and institutional accommodation did not have equal access to dental care [43].

Oral health behavior is a key factor at the population level for a healthy mouth. Tooth cleaning behavior (time and duration, frequency per day) has improved over the last thirty years. Oral hygiene at home is significantly supported by professional tooth cleaning in the practice and regular check-ups by the dentist, e.g., (re-)motivation and patient-specific instructions. For example, in Germany, control-oriented uptake is a well-established term, which refers to the fact that a patient in a preventive system regularly visits the dentist for check-ups even without dental problems. The value of a regular intake is recognized by these people. In contrast, dental care shifts in the later phases of life to problem-oriented dental care, which means that patients only come to the dental practice if they have oral health problems. This control-oriented usage behavior, which many people use and which has grown strongly over the years, (65–74-year-olds DMS III-1997: 57.1%, DMS IV-2005: 72.5%, DMS V-2014: 89.6%) is, thus, also to be considered a value for the people from the point of view of the population. Only 10.4% of the younger seniors visit the dentist with complaints. The older seniors (DMS V: control-oriented use 75–100 years 61.6% (*n* = 1127), 75–84 years 65.6% (*n* = 848), 85–100 years 49.3% (*n* = 280)) behave differently. In this group of 75–100-year-olds, 68.2% of the older seniors not in need of care were still showing a control-oriented uptake of dental services. Out of 253 study participants who were dependent on care, only 38.8% have seen a dentist for a check-up. In this group, it is also noticeable that only 33.3% of the nursing home residents choose or have a control-oriented approach to the dentist. The results in Table 1, Table 2 and Table 3 show that various factors are likely to change the previously controlling-oriented behavior and that a rearrangement of the personal value is not the only reason for the changes [43] resulting in the developed influencing-factor mechanism of the utilization of healthcare services.

Protective factors of utilization, which are patient-specific factors for the self-motivation of a control-oriented utilization of dental care, and factors that promote oral-health-related resilience and contribute to the improvement of control-oriented utilization of dental healthcare have been described in this paper. These are assumptions and theses of the expert panel and should, therefore, be evaluated regarding their evidence.

In addition, an attempt has been made to adapt or transfer terms and concepts for the field of gerodontology. Since gerodontology is a complex area in terms of ethical aspects [10], available terms cannot be transferred without adaptation. However, it is necessary to create and make use of concepts and terms in regard to further research in this area. The original meaning cannot always be completely fulfilled. The initial interpretation of the terms and concepts was considered in the further development and adapted to the requirements of gerodontology and clinicians working in this field.

### 4.2. Health as Part of the Ageing Policy

A society can function well if there is a balance between personal self-responsibility and independence on the one hand and social co-responsibility and “collective preventive and redistributive responsibilities” on the other [49]. This is also true in the area of risk management for the utilization of dental healthcare services: of course, each patient is individually and independently responsible for the maintenance of their own oral health. At the same time, the influence on the social and community level (Table 3)—especially in old age—should not be underestimated.

In this context, the duty of public welfare should also be mentioned, which is derived from the constitutional principle of the welfare state. This does not imply any direct entitlement of the citizen to benefits from the state, but it does mean that the state contributes to social security and justice by creating laws. Additionally, at the mesio level, health insurers have a duty of care towards their insured persons (e.g., in Germany: § 1 SGB V Solidarity and Personal Responsibility). This includes the task of providing and supporting “healthy living conditions” by means of education, counselling and direct services in order to “maintain or restore the health of the insured or improve their state of health” [50].

Part of the individual risk of a reduced utilization of dental healthcare services at the patient level is due to the patient’s subjective attitude to their own health. Studies have shown that the subjective assessment of oral health is better than that evaluated by a dentist [51]. This self-concept could be triggered in a targeted manner by continuous strengthening of the patient self-image (e.g., through health education, motivation, oral hygiene education, etc.) already beginning in early adulthood. It could be shown that a positive self-concept enables people to age in a health-promoting (salutogenetic) way [49]. Kruse points out that the ability of humans to cope with severe physical impairments should not be defined by the term resilience alone. The “sound medical and nursing care of the person as well as their social support” is indispensable [52]. For this purpose, health policy interventions are necessary but also conceivable in dentistry. Apart from long-term care facilities, older people are also often cared for in hospitals and in short-term care. This time should be used to enable the older person to undergo dental screening, thus increasing equality of opportunity and equity in access to dental services. At present, a detailed geriatric assessment is carried out in all areas (e.g., medicine, psychology, living situation). However, the oral cavity is not considered in the medical part. Further treatment after the short-term care could then be organized in the future place of residence in the framework of a consultation offer by the dentist and the caregivers; relatives could be informed.

Consistent legislative intervention at the interfaces may contribute to enabling older people to increase their resilience (in the sense of psychological resilience) and to utilize individual potential for prevention [49]. Therefore, it makes sense to develop and establish concepts, also for the interfaces to other disciplines, which may contribute to an improved development of the self-concept. It can be assumed that in this way, resources and resiliencies in old age will be better used in order to maintain the utilization of (dental) medical services.

### 4.3. Proposals for a Strategy for the Implementation of a Supportive Resilience Structure

Factors that promote the use of dental healthcare, support the older patient’s loyalty to the dental office and ensure the older person’s right or opportunity to receive dental treatment despite adverse living conditions (ensuring equity of care, opportunities to access (dental) medical services) are the basis of the oral-health-related resilience concept. The equity of care should be derived from medical–ethical principles. In the four-principles model according to Tom Lamar Beauchamp and James F. Childress [9], the principle of justice demands a decent, fair and just attitude in the rules of living together—i.e., in the behavior towards others—appropriate distribution of healthcare services while respecting resources. Similar or, of course, identical circumstances among patients should be treated equally, although this may cause difficulties in the need for support in the utilization of dental services, given the heterogeneity of older people as a patient group. Unequal treatment should be concretized according to ethical criteria, and any ethical dilemmas that may arise should then be specifically analyzed with the help of the four-principles model.

In order to enable older people, access to dental healthcare services, not only behavioral preventive interventions but also interventions of proportional prevention, must be activated. Behavioral preventive interventions relate directly to the individual person and, thus, to the individual health behavior, which must be strengthened. This includes, for example, interventions that strengthen one’s own health competence, but also one’s own ability to implement the desire to visit the dentist despite multimorbidity and restrictions in mobility. The aim is to reduce risk factors due to misbehavior (e.g., consequences of inadequate oral hygiene, no regular visits to the dentist, etc.). Proportional prevention considers, among other things, the residential and financial situation in which the older people find themselves. Here, it is important to introduce a fair distribution of resources in families, old people’s homes and long-term care facilities supported by the government and health insurance companies. The aim is to ensure that patients have access to regular dental healthcare throughout their lives, regardless of their circumstances, and to support this process. Factors and processes have to be identified, presented (including presentation of the criteria, advantages and disadvantages) and optimized, which aim to maintain the use of dental healthcare services despite a changed or adverse life situation (strengthening of oral-health-related resilience). This process, also known as primary prevention, also referred to as health promotion interventions, aims at eliminating risk factors before their effects occur [53]. Secondary prevention, on the other hand, refers to interventions for “early detection and early treatment” [53]. Here, for example, targeted prevention methods for risk groups (e.g., people diagnosed with dementia, people with disabilities) need to be considered. Tertiary prevention, on the other hand, would be the active intervention and treatment of existing health problems in order to prevent the disease from progressing any further [53] (e.g., rehabilitation of the diseased oral cavity and reintegration into a control-oriented, individualized dental care concept for the patient).

In order to ensure continued utilization of dental healthcare services by the older person for as long as possible, it is essential to balance risk and protective factors. In principle, the assumption can be made that in the case of the older person with more risk factors (e.g., a reduced therapeutic capability and self-responsibility in terms of the oral functional capacity) [12,19] the protective factors need to be even stronger (e.g., take-over of responsibility by the patient’s environment). However, since it is not always possible to adjust all factors to each other, the establishment of a supportive resilience structure aimed at generating an environment that secures the patient’s needs would be useful. This would allow handling even complex patient situations (multifactorial and multifinalite factors). However, individual resilience factors can have opposite effects, depending on the phase of life, biography and internal control behavior, whereby they can act both as risk factors and as protective factors. Therefore, the context and the situational circumstances should never be disregarded. The adaptability of a dental healthcare system for older people (dental case management) is mainly based on the possibility of activating resources: a positive attitude of the dental network, its active adaptability and a resource-efficient use of back-up capacities are the basis for this. Back-up capacities are to be found both in the patient’s environment (e.g., through a patient-friendly design of a dental office website), in the medical-care context (e.g., through the involvement of relatives or outpatient/inpatient care in the organization of dental appointments) and in the environment of the dental office (e.g., senior-friendly design of the dental office, development of individual treatment strategies, gerostomatologically trained team). These factors may be used as positive indicators (in comparison to the negatively weighted barriers to utilization) to objectively measure the utilization-related resilience of a care system.

Resilience-related research on utilization needs to focus less on the analysis of factors of emergency-oriented utilization or the development of prevention-oriented utilization than on securing and maintaining utilization despite adverse circumstances (favoring control-oriented utilization). Therefore, there is the possibility to utilize the endogenous resilience factors (e.g., to rely on the fact that relatives of a person suffering from dementia will also think about a visit to the dentist) or to create external conditions for realization (possibly with targeted personnel and material interventions) in order to promote a resilient utilization process (e.g., through a media). Due to the tendency of reduced endogenous capacities of the patient at an advanced age (e.g., increasing effort to independently organize an appointment to see the dentist) and also of the patient’s environment (e.g., possibility of relatives to accompany the elderly person to the dental office), above all exogenous resistance capacities (e.g., reimbursement of transport costs) and social support (e.g., organization of transport by nursing staff, but also age-appropriate treatment of the older person in the dental office) play a role. These resilience potentials are particularly important for older people and can be activated by considering and knowing the social–structural living conditions and lifestyles of older people. The more precisely the groups and their risks are evaluated, the better the support for equal opportunity access may be.

### 4.4. Need for Future Research

Future research should aim to eliminate the research gap, in particular by describing the resilience processes, determinants and modes of action that can secure medical care, as exemplified here by the visit to the dentist, even in old age. The concepts conveyed here, related to a sub-area of medical care, are intended to help to further develop the topic, i.e., also to motivate to work on it for other specialist areas, in order to point out the inequalities and age discrimination/ageism in access to medical services for the older population and to jointly develop solutions. The influencing and protective factors mentioned above should be methodically developed. For this purpose, quantitative elements (e.g., questionnaires) and also qualitative elements (e.g., interviews) can be used. It is also essential to include the target group—the vulnerable patients. An evaluation should be carried out both as a cross-sectional study and as a longitudinal study, since effects of utilization or non-utilization can in part only be expected in the later course. The results of the evaluation must be expertly evaluated, assessed with ethical expertise and made available to the public discourse.

## 5. Conclusions

Influencing and protective as well as modifying factors (e.g., vulnerability, resilience, sense of coherence) in relation to the utilization of dental services by seniors at different levels of the healthcare system have been identified. Terminological imprecision in the use and transfer of existing terms (sense of coherence, resilience, salutogenesis) to gerodontology were clarified, and a terminology for the usage in gerodontology was proposed. Achieving social and individual appreciation and establishing a prevention-oriented approach to utilization will be increasingly challenging, since the population is getting older and access is not equal in terms of opportunity. Resistance forces need to be strengthened in an ethical context. Studies should increasingly present resilience processes, determinants and modes of action at the various interfaces in the healthcare system, which can ensure sustainable medical care in old age.

## Figures and Tables

**Table 1:** Static (not individually influenceable) and dynamic (easily changeable) influencing factors, organized with magnitude of importance within the different levels of the healthcare system, which are able to model the risk of a reduced utilization of dental healthcare services (modeled after Nitschke et al., 2015 [8]).

Levels	Parties	Static Factors	Dynamic Factors
**Macro Level** (social-political level)	Population	- Laws - Compulsory health insurances ▪ Legal insurance obligation for dental treatment; basics of the fee schedule	- Policy priorities (e.g., climate, education, health) by changing governments - Laws/regulations [26,27,28,29] (e.g., cost separation between outpatient and inpatient care, access to treatments (cross-border healthcare [30]) - Distribution of resources among individual households
**Mesio Level** (social level)	Population Non-healthy peopleHealth-insured people	- Implementation regulations of the health insurers ▪ Subsidies for benefits for regular dental visits, fee regulations, etc. - Determinations of Joint Committees ▪ Health insurance, associations of panel dentists, patient counselling etc. - Offers of interdisciplinary cooperation	- Availability of dental healthcare services [20] - Economic situation of funding institutions/health insurance - Financability of treatment by patient/insurance for dental services [6,31] - Regional differences within a country/between countries [32,33,34]
**Micro Level** (individual patient–physican level)	Patients	- Subjective attitude towards, importance, value and estimation [6] of one’s own health - Support from third parties ▪ Family/relatives/volunteers ▪ Caring/therapeutic professional groups/general practitioner - Socio-demographic factors ▪ Sex, education, marital status, ethnicity/nationality, family size, retirement [5,7] - Socio-historical factor [23]	*Objective influencing factors* - Health status [7] ▪ Multimorbidity [5], frailty, degree of care, diagnosis of dementia, pain, limted mobility [5] (accessibility of dental office), polymedication ▪ Oral functional capacity/resilience capacity level [12] - Oral factors ▪ Tooth status/-loss, pain, dentures [5,7,13,14] - Health determinants ▪ Lifestyle [15], smoking, activity, diet, etc. [6] - Socio-demographic/-economic factors ▪ Age [5,7], income/wealth [7], living situation [5], change in residence/hospitalization *Subjective, rational reasons and factors* - Anxiety/dental anxiety [5,16,17] - Self-perception of the need for treatment [12,17,18] - Financial resources [17,19,20] for oral health/shame with financial shortages - Subjective cost estimation for gained health benefits - Individual right to neglect [21], rejection of social standards [22] - Refusal of teaching opinion of school medicine [22]
	Dentist	- Beginners vs. expert knowledge in gerodontology - Life circumstances (e.g., living together with elderly people, accompanying elderly people at the end of their lives, own upcoming retirement) Age/sex of the dentist	- Dentist and staff itself ▪ Expert knowledge in gerodontology, valuing older patients as a patient group, senior-adapted quality of dental treatment, one’s own attitude to health, diseases and age, negative experiences [24], etc. ▪ Lack of consideration of the oral functional capacity - Infrastructure and equipment ▪ Poor accessibility, lack of aids, lack of mobile dental care, etc. [5]
	Patient–Dentist	- Dental office has been located at the site for a long time	- Patient–dentist relationship [20,25] ▪ Rude staff ▪ Worse social interaction between dentist and staff ▪ Patient and time management (e.g., unpunctual start of treatment) ▪ No possibility for participation of the patient/relative (empowerment, shared decision making) - Change in an existing patient–dentist relationship because of changes in infrastructure or staff, lack of mobile equipment - Short-term patient–dentist relationship

**Table 2:** Protective factors of utilization—patient-specific factors for the self-motivation of a control-oriented utilization of dental care (listing and organization with magnitude of importance based on expert panel).

	Protective Factors of Utilization—Patient-Specific Factors
**Individual Level** **(Micro Level Patient)**	- Available self-responsibility, self-management ▪ State of a successfully self-motivated control-oriented utilization behavior over the last two years ▪ Stress management strategies (strengthened resilience by a sense of coherence) - Recognition of treatment and care needs - Awareness that oral health contributes to general health - Positive basic attitude towards general/oral health, the dentist/dental office - Willingness to use available financial resources for the maintenance of oral health

**Table 3:** Protective factors of utilization—factors that promote oral-health-related resilience and contribute to the improvement of control-oriented utilization of dental healthcare (listing and organization with magnitude of importance based on expert panel).

	Protective Factors of Utilization—Others Than Patient-Specific Factors
**Individual Level** **(Micro Level Dentist)** Dentist/team dependent qualities	**Gerostomatological “feel-good factor” of the dental office** - (Evidence of) expert knowledge in gerodontology (dentist, dental team including dental technician) ▪ Treatment according to the oral functional capacity of the patient ▪ Ethically plausible decisions even in the case of ethical dilemmas ▪ Availability of participatory decision-making process together with patients and supportive people ▪ Identification of patients’ additional support needs ▪ Training of the individual caregiver in terms of oral and denture hygiene - Soft skills ▪ Intercultural and language skills ▪ Empathic team with individualized duty of care (e.g., intercultural and language skills) ▪ Respectful and caring interaction with the family/relatives of the elderly patient - Infrastructure and Equipment ▪ Availability of equipment and aids suitable for older patients and a safe access to the dental office ▪ Availability of support in case of administrative barriers o Organization of the financing (e.g., clarification with health insurance companies, involvement of relatives/legal guardian early in the processes, keeping the dental bonus booklet) o Organization of transportation with the long-term care facilities o Organization of the list of diagnosis/medication from the medical doctor ▪ Complete integration of seniors in the dental office’s recall program (e.g., re-organization of new appointments with the dentist in case of a missed/cancelled appointment)
**Social Level** **Internal support level** Internal, social support by family/relatives and the population	- Support of the consultation visit and participatory decision-making process by family/relatives - Support or takeover of oral hygiene by trained third-party (non-) professional caregivers - Influence of generational attitudes towards oral health: strengthening of the relevance of lifelong utilization and care by relatives for those who are no longer responsible for their own (oral) health - Appreciation of the supporters of the older person
**Social Level** **External support level** External, social support (caregivers/volunteers/physicians)	- Inclusion of people in need of care as a vulnerable patient group in the regulations and laws (e.g., German SGB V), implementation of further (dental) medical services that this patient group needs in contrast to others - Education and training of caregivers to third-party oral-healthcare givers and oral-healthcare managers - Competent advice considering the dental needs in the long-term care facilities - Reduction in administrative barriers - Assumption of responsibility for oral health, support or takeover of oral hygiene by trained third-party (non-) professional caregivers (caregiver/oral healthcare manager in a long-term care facility) - Timely referral by physician to dentist - Establishment of treatment options in older people with reduced resilience or reduced mobility ▪ Organization of dental office visits (e.g., mobility services of foundations or non-profit-making associations) ▪ Offer of possibilities for further dental healthcare in the event of a change in residence (e.g., moving into a care facility) ▪ Offer of mobile dentistry by long-term care facilities and gerodontologists ▪ Careful handling of the elderly person, considering functional resilience ▪ Increased intercultural opening of existing offers, creation of transparency of offers - Result evaluation of the quality of healthcare providers - Target group specific design of counselling services (e.g., dental case and care management of the statutory health insurance companies) - Targeted public relations work to inform those affected and their relatives
**Social–Political Level**	- Duty of care of the state and health insurance - Organization of healthcare services at the health policy level - Senior-friendly intention of the legislator - Availability of social resources - Identify specific needs in the population (e.g., people living alone and in need of care and with a migration background) and approaches to meet them - Adequate financial security of care for oral diseases - Set of values of a society (are older people appreciated and, therefore, supported?) - Promotion of gerodontology by professional associations - Compulsory training in gerodontology with visits to long-term care facilities - Introduction of preventive home visits with integration of a dental module - Improvement of the access for vulnerable groups with a migration background

## Data Availability

No new data were created or analyzed in this study. Data sharing is not applicable to this article.
